# Risk of Metabolic Syndrome among Middle-Aged Koreans from Rural and Urban Areas

**DOI:** 10.3390/nu10070859

**Published:** 2018-07-03

**Authors:** Seohyun Lee, Yoonjin Shin, Yangha Kim

**Affiliations:** Department of Nutritional Science and Food Management, Ewha Womans University, Seoul 03760, Korea; hayeeun@empas.com (S.L.); yjin19@hotmail.com (Y.S.)

**Keywords:** metabolic syndrome, urban, rural, carbohydrate, sodium

## Abstract

Metabolic syndrome (MetS) is a common global health problem. This study aims to assess nutrient intake and risk of MetS in middle-aged Koreans based in residential areas. Participants were 161,326 (142,137 in urban and 19,189 in rural) subjects enrolled in the Korea Genome and Epidemiology Study. The prevalence of MetS was much higher in rural (39.8%) than that in urban (22.5%) subjects (*p* < 0.001). The rural residents showed significantly higher blood pressure (*p* < 0.001), serum triglyceride levels (*p* < 0.001), and LDL (Low density lipoprotein)-cholesterol level (*p* < 0.001), as well as the odds ratio (OR) for MetS (OR = 1.65, 95% CI: 1.59–1.71), compared to urban residents. The rural subjects showed a higher consumption of carbohydrate and sodium compared to the urban subjects (*p* < 0.001). After adjusting for potential confounders, subjects in the highest quartile of carbohydrate intake had higher OR for MetS (OR = 1.23, 95% CI: 1.15–1.32) and those in the highest quartile of sodium intake had a higher chance of having MetS (OR = 1.11, 95% CI: 1.07–1.16) than did those in the lowest quartiles. Our results suggested that the higher consumption of carbohydrate and sodium in rural residents might be associated with the increased risk of MetS in this population.

## 1. Introduction

Metabolic syndrome (MetS) is a major emerging global health problem. MetS is associated with the development of cardiovascular disease (CVD) and diabetes because it is primarily associated with abdominal obesity, high blood glucose, high blood pressure, and dyslipidemia. CVD is a main cause of mortality in Korea, and MetS is an important risk factor for CVD. The number of people with MetS is increasing at an alarming rate worldwide. Within the United States, 23.7% of the population had high prevalence of MetS, according to the definition of the National Cholesterol Education Program (NCEP) Adult Treatment Panel III (NCEP-ATP III) [1]. The number of individuals with MetS has also increased with the increase of abdominal obesity and dyslipidemia in Korea [2]. The incidence of age-adjusted MetS among participants in the Korean National Health and Nutrition Examination Survey (KNHNES) from 1998 to 2007 reflected a steady climb as shown by the following numbers: 24.9% in 1998, 29.2% in 2001, 30.4% in 2005, and 31.3% in 2007 [2]. The prevalence of MetS has increased in Korean adults of all ages, especially after middle-age [2].

Environmental factors, including urbanization, and westernization of lifestyle can significantly affect the prevalence of MetS among various population groups. MetS is affected by not only socioeconomic factors as education and income but also lifestyle factors such as diet, smoking, alcohol consumption, and physical activity [3]. Diet in particular has been reported to be strongly associated with the risk of MetS. Diets rich in fruits, vegetables, and whole grains have been reported to help prevent MetS [4], whereas meats, fried foods, and diet soda were adversely linked with incidence of MetS [5]. In addition, dietary nutrients such as carbohydrates, protein, fat, and sodium have been reported to be related to the prevalence of MetS [3].

Socioeconomic changes and urbanization throughout their economic growth eventually affected a nutritional transition within developing Asian countries such as South Korea [6], China, Malaysia, and India [7]. Urbanization is related to increased intake of energy-rich foods and shift to a Western-style diet. However, these changes generally occur at prominently different rates across urban-rural residential areas. The dietary habits of urban residents are characteristically high in sugar, fat, and animal protein. Residents of rural areas, however, have generally retained a more traditional diet of natural foods than those in urban areas [8]. Rapid socioeconomic transition in Korea over the last several decades brought significant change in the lifestyle of the people, resulting in changes in MetS prevalence patterns. Lifestyle and diet, major factors affecting MetS, are reported to be different in urban and rural areas in Korea [9,10].

To better understand the prevalence of MetS of urban and rural areas in Korea, large-scale epidemiologic studies of populations across a wide range of communities are needed. Furthermore, it is meaningful to examine regional characteristics and the MetS prevalence according to the residential areas through large-scale cohort data because it could be useful as basic data to inform the development of public health promotion programs and related policies for prevention or reduction of MetS in each region. The purpose of this study was to investigate nutrient intake and the risk of MetS according to rural and urban areas using data of the Korea Genome and Epidemiology Study (KoGES).

## 2. Materials and Methods 

### 2.1. Data Source and Study Population

Baseline examination data from the Health Examinee (HEXA) cohort and Cardiovascular Disease Association Study (CAVAS) cohort, two subprojects of KoGES were used in this study. The KoGES is an ongoing population-based cohort to investigate genetic and environmental risk factors and their interaction with the main diseases found in the Korean population. The baseline examination of the HEXA cohort and CAVAS cohort consist of urban- or rural-community dwellers, respectively. The baseline investigation of the HEXA cohort was performed at 38 community-health examination centers and training hospitals located in 14 large urban areas during 2004–2013, and the CAVAS cohort was conducted at 11 rural areas during 2005–2011 in Korea. The participants, who were aged 40–70 years at baseline, were recruited from the national health examinee registry. We used their baseline survey and measurement data. More detailed information on the aims, the community characteristics, and the baseline characteristics is given elsewhere [11]. All participants completed an interviewer-administered questionnaire that included sociodemographic and behavioral characteristics and a validated food frequency questionnaire. Anthropometric measurements and biochemical measurements were also performed for all participants. Informed consent was obtained from all participants.

Initially, 201,695 subjects (173,357 in urban and 28,338 in rural) were recruited at baseline. We exclude participants who reported implausible daily energy intake (<800 or >4000 kcal/day in men, <500 or >3500 kcal/day in women), those over aged 64 years old, those who provided insufficient anthropometric information, and those who did not respond to biochemical information (total cholesterol, triglyceride, high density lipoprotein (HDL)-cholesterol, diastolic blood pressure (DBP), systolic blood pressure (SBP), fasting glucose level). After exclusion, 161,326 (142,137 in urban and 19,189 in rural) were included in the final data analyses. The study protocol was approved by the Ethics Committee of the Korean Health and Genome Study and the Institutional Review Board of the Ewha Womans University (IRB No:135-1), which was in compliance with the Declaration of Helsinki.

### 2.2. Dietary Assessment

Dietary intake data were collected through a semi-quantitative food frequency questionnaire (SQFFQ) involving 106 items, which was developed and validated by Korea Centers for Disease Control and Prevention (KCDC) for KoGES. More detailed information concerning the protocol and the results of a validation study about SQFFQ is given elsewhere [12]. Nutrient intake was measured using a database developed by the Rural Development and Administration of Korea.

### 2.3. Definition of Metabolic Syndrome

Metabolic syndrome was defined based on the National Cholesterol Education Program (NCEP) Adult Treatment Panel III criteria [13] as having three or more of the following components: (i) abdominal obesity: a waist circumference ≥90 cm in men and ≥80 cm in women (waist circumference criteria was modified by the International Obesity Task Force criteria for Asian-pacific populations [14]); (ii) high triglycerides level: ≥150 mg/dL (1.70 mmol/L); (iii) low HDL-cholesterol level: <40 mg/dL (1.04 mmol/L) in men and <50 mg/dL (1.30 mmol/L) in women; (iv) elevated blood pressure: ≥130/85 mmHg or antihypertensive medication; (v) elevated fasting blood glucose: ≥100 mg/dL (5.60 mmol/L) or medication (insulin or oral agents).

### 2.4. Other Measurements

Demographic characteristics, socioeconomic status, and health-related factors data were collected using a standardized questionnaire. Education status as present socioeconomic status was categorized into four groups: ≤Elementary school, ≤High school, ≤University, >University. Monthly income level was divided into four groups: <$1000, $1000–2000, $2000–4000, >$4000. Smoking status and alcohol consumption were classified as current or past/never. Regular exercise was classified as “yes” or “no” depending on whether participants regularly exercised enough to sweat at least once a week. Marital status was categorized by married or other, including single, separated, divorced, widowed, and cohabiting. The presences of CVD and cancer were assessed by self-reporting. Myocardial infarction, angina pectoris, and stroke were considered CVD. 

Anthropometric characteristics were also collected using standardized methods. Height, weight, waist circumference, and hip circumference were collected using standardized techniques and calibrated equipment. Height and weight were measured to the nearest 0.1 cm or 0.1 kg, respectively. Body mass index (BMI), which reflects obesity status, was defined as weight (kg)/height (m^2^). Participants were classified into two categories based on BMI: <25 kg/m^2^ or ≥25 kg/m^2^. Waist circumference was measured at the narrowest point between the lowest rib and the right iliac crest to the nearest 0.1 cm. Hip circumference was measured at the maximal extension of the buttocks to the nearest 0.1 cm. Blood pressure was recorded after participants had rested for more than 10 min. SBP and DBP were measured two times in the right arm using an automatic sphygmomanometer or standardized mercury depending on the institution. Blood total cholesterol, HDL-cholesterol, triglyceride and glucose levels were measured using the enzyme method (ADVIA 1650 and ADVIA 1800; Siemens Healthineers, Deerfield, IL, USA). Low density lipoprotein (LDL)-cholesterol level was calculated by using the Friedewald formula [15] in individuals with blood triglyceride levels <400 mg/dL (<4.52 mmol/L).

### 2.5. Statistical Analysis

Sociodemographic characteristics and health-related variables are expressed as means with their standard error (continuous) or number with percentages (categorical). Differences in baseline characteristics were examined by Student’s *t* test for continuous variables or chi-square test for categorical variables by dwell region and sex. The general linear model was used to examine for significant differences in mean values for nutrient intakes. Multivariable logistic regression analysis was used to assess to comparison of the risk of MetS between rural and urban areas. The 95% confidence intervals (95% CI) of the odds ratio (OR) were estimated using the Wald method. Model I was crude data. Model II was adjusted for age, education level, household income, smoking status, alcohol intake, regular exercise, BMI, and daily total energy intake. In this study, BMI was considered as a potential confounder variable due to it being an important risk factor for MetS and its components. However, as BMI has a high correlation with waist circumference, the final model excluded BMI from OR calculation of abdominal obesity, a component of MetS. Logistic regression model was also used to analyze whether the multivariate-adjusted OR for MetS was associated with carbohydrate and sodium intake. The linear trend test across increasing categories of carbohydrate and sodium was conducted as continuous variables using median consumption of within each category. All the data were analyzed using SAS 9.4 (SAS Institute, Cary, NC, USA). All of the *p*-values were two-sided, and statistical significance was defined as *p* < 0.05.

## 3. Results

### 3.1. General Characteristics of Study Population

The sociodemographic, anthropometric, clinical, and biochemical variables of the study subjects are shown in Table 1 and Table 2. The mean age for rural men and women was higher than that of urban men and women, and the proportion of population over 50 years old was significantly higher in rural areas than in urban. The rural participants had lower rates of education and lower household incomes. The proportion of the male smokers was higher in rural than urban locations, but there were fewer female smokers in rural than in urban areas. The percentage of alcohol intake and regular exercise in rural populations were lower than those of urban dwellers. 

Within the physical and blood biochemical parameters, overall, rural subjects had higher weight, waist circumference, SBP, and DBP than their urban counterparts. BMI was higher in rural women than urban women, but not men. Fasting blood glucose, levels of triglyceride, total cholesterol, LDL-cholesterol, and the ratio of LDL-cholesterol to HDL-cholesterol were higher in rural dwellers compared to those of urban populations. On the other hand, the HDL-cholesterol level was lower in rural people than in urban people. 

### 3.2. Nutrients Intake of Study Population

Table 3 shows the subjects’ daily nutrient consumption by residence and gender. Total daily energy intakes of all participants were 1697.7 kcal for rural subjects and 1750.8 kcal for urban subjects. Rural residents had a significantly lower energy intake compared to their urban counterparts. Macronutrient intakes, including protein and fat, were also significantly lower in rural than in urban areas. The ratio of carbohydrate to protein to fat displayed a similar pattern without reference to residence. However, the individual ratios of macronutrients showed differences between urban and rural areas. The carbohydrate portion for total energy was significantly higher in rural populations than in urban populations; however, urban subjects consumed more protein and fat than rural subjects. Most micronutrient consumption was significantly lower in rural residents than those of urban residents, except for sodium intake; which was significantly higher for both genders in rural areas than that of the urban areas. 

### 3.3. Urban-Rural Comparision of Metabolic Syndrom

Table 4 shows the overall prevalence comparison of MetS by NCEP Asian-Pacific criteria. The prevalence of metabolic syndrome was significantly higher in rural populations than urban populations. The prevalence of MetS was 39.8% among rural residents and 22.5% among urban residents. Prevalence of the MetS components by gender and residence place is also shown in Table 4. The prevalence of the MetS components showed similar results to the prevalence of MetS between rural and urban populations. Of all subjects, crude prevalence of MetS components was higher in rural participants than those of urban participants. High blood pressure was the most common factor of MetS in men (52.0% in rural and 48.6% in urban), but low HDL-cholesterol was the most common component in rural women (65.2% in rural and 31.4% in urban), followed by abdominal obesity (62.2% in rural and 40.8% in urban). Men had a higher prevalence of high blood pressure, high triglyceride, and high blood glucose factors than women, whereas women had a higher prevalence of low HDL-cholesterol and abdominal obesity than men. Table 4 presents the OR for MetS in rural and urban areas. The crude OR for MetS was significantly higher in rural populations (OR = 2.26, 95% CI: 2.19–2.33) compared to the urban subjects. The overall crude OR for all the individual components of MetS were also significantly higher in rural subjects than in urban residents. Even after multivariate adjustment, urban-rural areas were associated with OR for MetS and MetS components such as abdominal obesity, high triglyceride, and low LDL-cholesterol. 

In this study, higher intake of carbohydrate or sodium was associated with increase of OR for MetS. Table 5 presents the OR for MetS by quartile of carbohydrate and sodium consumption. The crude OR for MetS was significantly higher in the highest carbohydrate (OR = 1.07, 95% CI: 1.01–1.13, *p* for trend 0.003 in men; OR = 1.09, 95% CI: 1.05–1.14, *p* for trend <0.0001 in women) and sodium (OR = 1.20, 95% CI: 1.14–1.27, *p* for trend <0.0001 in men; OR = 1.14, 95% CI: 1.10–1.19, *p* for trend <0.0001 in women) consumption category than those in the lowest carbohydrate and sodium intake category. After adjusting for potential confounder variables, the overall OR for MetS also increased with the highest consumption of carbohydrate (OR = 1.23, 95% CI: 1.14–1.33, *p* for trend <0.0001) and sodium (OR = 1.11, 95% CI: 1.06–1.16, *p* for trend <0.0001) compared to those in the lowest category of carbohydrate and sodium consumption. 

## 4. Discussion

The present study investigated nutrient intake status and prevalence of MetS in urban and rural populations of middle-aged Koreans using two large cohort studies. To examine the characteristics of the populations between urban and rural areas, we analyzed the sociodemographic parameters, anthropometric measurements, blood biochemical parameters, and nutrients intake of the subjects of each region. The rural subjects were older than the urban subjects. It has been reported that the average age of Koreans is higher in rural areas than urban areas [9,10,16], and the proportion of the aging population is also higher in rural than urban locations [10]. The age of the members of a society is one of the most important determinants of socioeconomic change and could affect various characteristics, such as household income of the society. In the current study, level of education and household income, as well as sociodemographic characteristics, were significantly lower in rural residents than urban people for both genders. These results were consistent with the results of a study on urban and rural areas of people older than 55 years old in 13 provinces of Korea [10]. 

Metabolic syndrome is an important public health concern in Korea, and the prevalence of MetS among Koreans has been increasing [2]. Global prevalence of MetS in the adult population is approximated to be 20–25% [17] and the prevalence is estimated to be 11.9–37.1% in Asia-Pacific countries [18]. Our results showed that the prevalence of MetS in urban populations was similar to the global average. However, the proportion of MetS prevalence for people in rural areas was noticeably higher, about 40%, which is the highest level among Asia-Pacific countries [18]. It has been reported that the annual cost of healthcare is associated with chronic diseases [19]. According to the Korea Institute for Health and Social Affairs report, the annual cost of the healthcare for MetS-related diseases was about 3.8% higher in rural areas compare to urban areas in 2011 [20].

The MetS prevalence generally occurs at significantly different rates according to age, socioeconomic environment, residential area, and dietary nutritional status [3]. Aging is one of the well-known risk factors of MetS [3]. Our results showed that rural subjects were older than the urban subjects, and the MetS prevalence was higher in rural dweller than urban people. Li et al. [21] also reported that the prevalence and the risk for MetS were higher in older adults in China. Low socioeconomic status, as determined by the level of education and income [22], has been shown to be possibly linked with a higher prevalence of MetS [22,23]. A recent Chinese study showed that the prevalence and the risk for MetS were higher in those with low level of education in 2014–2015 [21]. Our results indicated an inverse relationship of education level or home economics to the prevalence of MetS. Rural residency indicated a relatively lower level of education and household income than urban populations and reflected a higher prevalence of MetS. Physical activity may be the crucial factor in the aetiology of MetS. Physical inactivity has an independent effect on the components of MetS [24]. It has been reported that vigorous and moderate physical activity is related to a reduced risk of MetS in white European people [25]. Our findings showed that rural dwellers exercise less frequently than urban people.

Dietary nutrient intake has been an important indicator of metabolic disorders such as diabetes mellitus, obesity, dyslipidemia, and MetS [3]. In this study, there were differences in intakes of nutrients such as protein, vitamins, and iron between rural and urban areas. The intake levels of nutrients except carbohydrate and sodium were lower in rural populations compared to urban populations. The carbohydrate and sodium were consumed in significantly higher amounts by rural dwellers. Thus, we focused to analyses the OR for MetS based on carbohydrate and sodium consumption. Carbohydrate-rich diets are known to be the major cause of aggravation of glucose intolerance [26]. In addition, high carbohydrate intake is known to be associated with lipid abnormalities, such as elevated triglyceride and reduced HDL-cholesterol levels [27]. We showed higher fasting blood glucose levels in rural dwellers with relatively high percentage of caloric intake from carbohydrate than in urban subjects. In blood lipid profiles, triglyceride and LDL-cholesterol levels were higher in people in rural areas compared to those in urban areas, but the HDL-cholesterol level was lower in rural subjects than urban people were. Moreover, we found that the risk of MetS was positively associated with increasing carbohydrate intake. The high proportion of carbohydrate intake is a major characteristic of the Korean diet because rice, a main source of carbohydrate, is the staple food for Koreans. Although the dietary habits of contemporary Koreans have changed due to urbanization and westernization, it has been reported that rural residents tend to retain a more traditional Korean diet consisting of carbohydrate-rich foods compared to urban people [10].

The present study showed that sodium intake in rural areas was higher than urban areas, and blood pressure was also higher in rural areas than in urban areas. A high-sodium diet has been related with various metabolic diseases such as hypertension and CVD [28]. High sodium consumption was also positively associated with increasing prevalence of MetS [29]. Our results showed that the prevalence of MetS in rural populations was higher than in urban people. In addition, OR for MetS also increased with increasing sodium intake. This observation is consistent with the results of sodium intake and the risk of MetS in Koreans [30]. Kimchi, soups, and stews were the main sources of sodium intake for the Korean population [31], and they are major elements of the traditional Korean food structure. According to a traditional Korean-diet study, rural people has higher proportion of a traditional diet pattern, consuming rice-based staple food and kimchi, than metropolitan people [32].

The higher prevalence of MetS in urban residents compared to the rural population have been reported in Eastern China [33], India [34], and Malaysia [35]. Conversely, other studies have reported that the prevalence of MetS in rural areas was higher [9,36]. Our findings showed that the prevalence and the OR of the MetS were significantly higher in rural populations than those in urban areas. The difference in the prevalence of MetS between urban and rural areas might be attributed to the difference in the demographic and sociocultural characteristics of those areas [37]; thus, the disparity of the Mets-related factors such as age, physical inactivity, and nutrient intake between urban and rural inhabitants in each country can affect different outcomes for the prevalence of MetS in rural and urban across countries. The urban people in Eastern China, reported to have the higher prevalence of MetS, showed higher consumption of fat with less physical activity compared to rural residents [33]. Our results showed that rural people consume more carbohydrates and sodium and less regular exercise than urban residents do.

Prevention is the most critical strategy to reduce the MetS and its outcomes. Improper nutrition and inadequate physical activity are the main causes of MetS, so health education to improve lifestyle may be an important part in establishing public health policy. To reduce the risk of MetS in Korea, diverse efforts by the community as well as various national health promotion programs are required. Our findings support the need for targeted efforts to develop and implement MetS prevention programs for urban and rural residents in Korea.

The present study was a cross-sectional analysis and thus has the limitation that a temporal relationship between individual nutrient consumption and prevalence of MetS could not be established. Future studies will require long-term follow-up studies of nutrient intake and MetS to better understand the direct causes of varying MetS prevalence in different regions in Korea. Another limitation of this study was that the participants were recruited from 11 rural and 14 urban communities to secure representative samples, but only volunteers took part in the study. Therefore, collected data has been contained selection bias between study participants and non-participants. Consequently, our findings should be applied carefully to the whole rural and urban Korean population. 

## 5. Conclusions

In summary, this study showed that the prevalence and risk of MetS in the middle-aged Korean population were higher in rural dwellers compared to urban populations. These differences by residential areas paralleled higher intake of carbohydrate and sodium in rural residents than urban dwellers. Moreover, the MetS-related factors such as education level, household income, and rate of regular exercise were lower in rural people than those of urban subjects. Our findings suggested that the difference in sociocultural environmental factors and lifestyle, such as nutrient intake, might partially contribute to the difference in the prevalence and risk of MetS between urban and rural populations across regions in Korea.

## Figures and Tables

**Table 1:** General characteristics of rural and urban Korean adults aged 40–64, CAVAS (2005-2011) and HEXA (2004–2013) cohorts.

	Men (*n* = 53,704)			Women (*n* = 107,622)			Overall (*n* = 161,326)		
	Urban (*n =* 46,680)	Rural (*n =* 7024)	*p-*Value	Urban (*n =* 95,457)	Rural (*n =* 12,165)	*p-*Value	Urban (*n =* 142,137)	Rural (*n =* 19,189)	*p-*Value
Age (year)	51.68 ± 0.03	54.53 ± 0.08	<0.0001	51.17 ± 0.02	53.65 ± 0.06	<0.0001	51.34 ± 0.02	53.95 ± 0.05	<0.0001
40–49	18,568 (39.78)	1749 (24.90)	<0.0001	39,806 (41.70)	3612 (29.69)	<0.0001	58,374 (41.07)	5361 (27.94)	<0.0001
50–64	28,112 (60.22 )	5275 (75.10 )	<0.0001	55,651 (58.30)	8553 (70.31)	<0.0001	83,763 (58.93)	131,828 (72.06)	<0.0001
Education level			<0.0001			<0.0001			<0.0001
≤Elementary	4051 (8.68)	2493 (35.49)		17,112 (17.93)	6761 (55.58)		21,163 (14.89)	9254 (48.23)	
≤High school	21,520 (46.10)	3470 (49.40)		54,381 (56.97)	4616 (37.94)		75,901 (53.40)	8086 (42.14)	
≤University	16,275 (34.87)	822 (11.70)		20,219 (21.18)	697 (5.73)		36,494 (25.68)	1519 (7.92)	
>University	4325 (9.27)	216 (3.08)		2613 (2.74)	60 (0.49)		6938 (4.88)	276 (1.44)	
Unknown	509 (1.09)	23 (0.33)		1132 (1.19)	31 (0.25)		1641 (1.15)	54 (0.28)	
Income (USD/month)			<0.0001			<0.0001			<0.0001
<1000	2275 (4.87)	1615 (22.99)		8484 (8.89)	3423 (28.14)		10,759 (7.57)	5038 (26.25)	
1000–2000	7074 (15.15)	1495 (21.28)		16,640 (17.43)	145 (15.99)		23,714 (16.68)	3440 (17.93)	
2000–4000	19,177 (41.08)	1180 (16.80)		35,205 (36.88)	1693 (13.92)		54,382 (38.26)	2873 (14.97)	
>4000	12,601 (26.99 )	365 (5.20)		19,946 (20.90)	507 (4.17)		32,547 (22.90)	872 (4.54)	
Unknown	5553 (11.90)	2369 (33.73)		15,182 (15.90)	4597 (37.79)		20,735 (14.59)	6966 (36.30)	
Marital status			0.176			<0.0001			0.657
Married	41,802 (89.55)	6299 (89.68)		79,355 (83.13)	10,061 (82.70)		121,157 (85.24)	16,360 (85.26)	
Others	3043 (6.52)	478(6.81)		12,018 (12.59)	1672 (13.74)		15,061 (10.60)	2150 (11.20)	
Unknown	1835 (3.93)	247(3.52)		4084 (4.28)	432 (3.55)		5919 (4.16)	679 (3.54)	
Smoking status			<0.0001			<0.0001			0.104
Past/never	30,757 (65.89)	4447 (63.32)		92,770 (97.19)	11,919 (97.98)		123,527 (86.91)	16,366 (85.29)	
Current	15,820 (33.89)	2572 (36.62)		2323 (2.43)	227 (1.57)		18,143 (12.76)	2799 (14.59)	
Unknown	103 (0.22 )	5(0.07)		364 (0.38)	19 (0.16)		467 (0.33)	24 (0.13)	
Alcohol intake			<0.0001			<0.0001			<0.0001
Past/never	11,801 (25.28)	2269 (32.30 )		63,785 (66.82)	8461 (69.55)		75,586 (53.18)	10,730 (55.92)	
Current	34,800 (74.55)	4747 (67.58)		31,345 (32.84)	3667 (30.14)		66,145 (46.54)	8414 (43.85)	
Unknown	79 (0.17)	8 (0.11)		327 (0.34)	37 (0.16)		406 (0.29)	45 (0.23)	
Regular exercise			<0.0001			<0.0001			<0.0001
No	20,463 (43.84)	4642 (66.09)		47,008 (49.25)	7941 (65.28)		67,471 (47.47)	12,583 (65.57)	
Yes	26,140 (56.00)	2363 (33.64 )		48,264 (50.56)	4206 (34.57)		74,404 (52.35)	6569 (34.23)	
Unknown	77 (0.16)	19 (0.27)		185 (0.19)	18 (0.15)		262 (0.18)	37 (0.19)	
Disease History									
CVD	10,441 (22.37)	1736 (24.72)	<0.0001	15,931 (16.69)	2897 (23.81)	<0.0001	26,372 (18.55)	4633 (24.14)	<0.0001
Cancer	838 (1.80)	110 (1.57)	<0.0001	3294 (3.45)	281 (2.31)	<0.0001	4132 (2.91)	391 (2.04)	<0.0001

CAVAS: Cardiovascular Disease Association Study; HEXA: Health Examinee; USD: United States dollar; CVD: Cardiovascular disease. Values are expressed as mean ± SE or as the number of cases (%); Categorical variables were analyzed using the χ^2^ test; continuing variables were analyzed using the *t*-test. Overall data was adjusted for sex using logistic regression for categorical variables and general linear regression for continuous variables.

**Table 2:** Anthropometric parameters, blood pressure, and blood profiles of rural and urban Korean adults.

	Men (*n* = 53,704)			Women (*n* = 107,622)			Overall (*n* = 161,326)		
	Urban (*n =* 46,680)	Rural (*n =* 7024)	*p-*Value	Urban (*n =* 95,457)	Rural (*n =* 12,165)	*p-*Value	Urban (*n =* 142,137)	Rural (*n =* 19,189)	*p-*Value
Height (cm)	169.02 ± 0.03	166.68 ± 0.07	<0.0001	156.60 ± 0.02	154.27 ± 0.05	<0.0001	160.74 ± 0.01	158.40 ± 0.04	<0.0001
Weight (kg)	70.02 ± 0.04	68.07 ± 0.11	<0.0001	57.89 ± 0.03	58.85 ± 0.08	<0.0001	61.93 ± 0.02	61.84 ± 0.06	0.146
BMI (kg/m^2^)	24.48 ± 0.01	24.46 ± 0.03	0.651	23.61 ± 0.00	24.72 ± 0.03	<0.0001	23.90 ± 0.01	24.60 ± 0.02	<0.0001
<25	27,586 (59.10)	4080 (58.09)	<0.0001	68,677 (71.95)	6978 (57.36)	<0.0001	96,263 (67.73)	11,058 (57.63)	<0.0001
≥25	19,094 (40.90)	2944 (41.91)		26,780 (28.05)	5187 (42.64)		45,874 (32.27)	8131 (42.37)	
WC (cm)	85.78 ± 0.03	86.34 ± 0.09	<0.0001	78.20 ± 0.03	82.61 ± 0.08	<0.0001	80.72 ± 0.02	83.74 ± 0.06	<0.0001
HC (cm)	96.14 ± 0.03	94.78 ± 0.07	<0.0001	93.56 ± 0.02	94.26 ± 0.06	<0.0001	94.42 ± 0.02	94.37 ± 0.04	0.332
Waist-hip ratio	0.89 ± 0.00	0.91 ± 0.00	<0.0001	0.84 ± 0.00	0.88 ± 0.00	<0.0001	0.85 ± 0.00	0.89 ± 0.00	<0.0001
SBP (mmHg)	125.34 ± 0.07	126.84 ± 0.20	<0.0001	120.22 ± 0.05	123.75 ± 0.16	<0.0001	121.92 ± 0.04	124.72 ± 0.11	<0.0001
DBP (mmHg)	78.81 ± 0.05	82.27 ± 0.13	<0.0001	74.64 ± 0.03	78.53 ± 0.10	<0.0001	76.03 ± 0.03	79.76 ± 0.07	<0.0001
FBG (mg/dL)	98.87 ± 0.11	102.49 ± 0.34	<0.0001	92.57 ± 0.06	95.54 ± 0.19	<0.0001	94.67 ± 0.06	97.87 ± 0.15	<0.0001
Triglyceride (mg/dL)	154.74 ± 0.52	173.27 ± 1.53	<0.0001	112.03 ± 0.24	135.98 ± 0.77	<0.0001	126.42 ± 0.24	148.25 ± 0.65	<0.0001
Total cholesterol (mg/dL)	194.25 ± 0.16	195.03 ± 0.43	0.088	199.28 ± 0.12	202.78 ± 0.34	<0.0001	197.60 ± 0.09	200.12 ± 0.26	<0.0001
HDL-cholesterol (mg/dL)	49.63 ± 0.05	43.40 ± 0.13	<0.0001	56.56 ± 0.04	46.70 ± 0.10	<0.0001	54.25 ± 0.03	45.71 ± 0.09	<0.0001
LDL-cholesterol (mg/dL)	115.25 ± 0.15	119.47 ± 0.39	<0.0001	120.61 ± 0.10	129.51 ± 0.30	<0.0001	118.86 ± 0.08	126.10 ± 0.23	<0.0001
LDL/HDL	2.42 ± 0.00	2.87 ± 0.01	<0.0001	2.23 ± 0.00	2.88 ± 0.01	<0.0001	2.30 ± 0.00	2.88 ± 0.01	<0.0001

BMI: Body mass index; WC: Waist circumference; HC: Hip circumference; SBP: Systolic blood pressure; DBP: Diastolic blood pressure; FBG: Fasting blood glucose; HDL: High density lipoprotein; LDL: Low density lipoprotein. Values are expressed as means ± SE or as the number of cases (%).Categorical variables were analyzed using the χ^2^ test; continuing variables were analyzed using the *t*-test. Overall data was adjusted for sex using logistic regression for categorical variables and general linear regression for continuous variables.

**Table 3:** Daily nutrients intake of rural and urban Korean adults.

	Men (*n* = 53,704)				Women (*n* = 107,622)				Overall (*n* = 161,326)			
	Urban (*n =* 46,680)	Rural (*n =* 7024)	*p* ^†^	*p* ^§^	Urban (*n =* 95,457)	Rural (*n =* 12,165)	*p* ^†^	*p* ^§^	Urban (*n =* 142,137)	Rural (*n =* 19,189)	*p* ^†^	*p* ^§^
Total energy intake (kcal/day)	1866.43 ± 2.32	1845.07 ± 6.17	0.001		1692.98 ± 1.61	1622.04 ± 4.29	<0.0001		1750.75 ± 1.32	1697.72 ± 3.59	<0.0001	
Carbohydrate (g/1000 kcal)	177.17 ± 0.08	182.10 ± 0.20	<0.0001	<0.0001	179.72 ± 0.06	187.44 ± 0.15	<0.0001	<0.0001	178.87 ± 0.05	185.58 ± 0.12	<0.0001	<0.0001
Protein (g/1000 kcal)	33.51 ± 0.03	31.70 ± 0.07	<0.0001	<0.0001	33.72 ± 0.02	31.30 ± 0.06	<0.0001	<0.0001	33.65 ± 0.02	31.45 ± 0.04	<0.0001	<0.0001
Fat (g/1000 kcal)	16.09 ± 0.03	14.39 ± 0.07	<0.0001	<0.0001	15.26 ± 0.02	12.45 ± 0.05	<0.0001	<0.0001	15.54 ± 0.02	13.13 ± 0.04	<0.0001	<0.0001
CHO% of energy	71.80 ± 0.03	74.01 ± 0.09	<0.0001	<0.0001	72.57 ± 0.02	76.00 ± 0.06	<0.0001	<0.0001	72.32 ± 0.02	75.31 ± 0.05	<0.0001	<0.0001
Protein% of energy	13.56 ± 0.01	12.87 ± 0.03	<0.0001	<0.0001	13.59 ± 0.01	12.67 ± 0.02	<0.0001	<0.0001	13.58 ± 0.01	12.74 ± 0.02	<0.0001	<0.0001
Fat% of energy	14.64 ± 0.02	13.13 ± 0.06	<0.0001	<0.0001	13.83 ± 0.02	11.32 ± 0.05	<0.0001	<0.0001	14.10 ± 0.01	11.95 ± 0.04	<0.0001	<0.0001
Calcium (mg/1000 kcal)	223.09 ± 0.43	211.66 ± 1.15	<0.0001	<0.0001	268.86 ± 0.38	246.54 ± 1.07	<0.0001	<0.0001	253.63 ± 0.30	235.25 ± 0.80	<0.0001	<0.0001
Phosphorus (mg/1000 kcal)	491.09 ± 0.41	476.52 ± 1.06	<0.0001	<0.0001	518.47 ± 0.34	495.83 ± 0.94	<0.0001	<0.0001	509.36 ± 0.26	489.64 ± 0.71	<0.0001	<0.0001
Iron (mg/1000 kcal)	5.38 ± 0.01	5.00 ± 0.02	<0.0001	<0.0001	5.87 ± 0.01	5.34 ± 0.02	<0.0001	<0.0001	5.70 ± 0.00	5.23 ± 0.01	<0.0001	<0.0001
Potassium (mg/1000 kcal)	1200.19 ± 1.69	1168.44 ± 4.53	<0.0001	<0.0001	1340.89 ± 1.43	1284.43 ± 4.22	<0.0001	<0.0001	1294.07 ± 1.12	1246.54 ± 3.04	<0.0001	<0.0001
Vitamin A (RE/1000 kcal)	257.34 ± 0.68	241.31 ± 1.89	<0.0001	<0.0001	281.75 ± 0.54	257.76 ± 1.58	<0.0001	<0.0001	273.63 ± 0.43	252.52 ± 1.17	<0.0001	<0.0001
Vitamin B1 (mg/1000 kcal)	0.58 ± 0.00	0.56 ± 0.00	<0.0001	<0.0001	0.57 ± 0.00	0.54 ± 0.00	<0.0001	<0.0001	0.57 ± 0.00	0.55 ± 0.00	<0.0001	<0.0001
Vitamin B2 (mg/1000 kcal)	0.49 ± 0.00	0.46 ± 0.00	<0.0001	<0.0001	0.53± 0.00	0.48 ± 0.00	<0.0001	<0.0001	0.52 ± 0.00	0.47 ± 0.00	<0.0001	<0.0001
Niacin (mg/1000 kcal)	8.26 ± 0.01	7.77 ± 0.02	<0.0001	<0.0001	8.29 ± 0.01	7.68 ± 0.02	<0.0001	<0.0001	8.28 ± 0.00	7.71 ± 0.01	<0.0001	<0.0001
Vitamin C (mg/1000 kcal)	52.29 ± 0.12	50.31 ± 0.32	<0.0001	<0.0001	66.21 ± 0.11	63.41 ± 0.32	<0.0001	<0.0001	61.57 ± 0.09	59.07 ± 0.23	<0.0001	<0.0001
Zinc (μg/1000 kcal)	4.50 ± 0.00	4.22 ± 0.01	<0.0001	<0.0001	4.53 ± 0.00	4.22 ± 0.01	<0.0001	<0.0001	4.52 ± 0.00	4.22 ± 0.01	<0.0001	<0.0001
Vitamin E (mg/1000 kcal)	4.38 ± 0.01	4.08 ± 0.02	<0.0001	<0.0001	4.73 ± 0.01	4.38 ± 0.02	<0.0001	<0.0001	4.61 ± 0.00	4.28 ± 0.01	<0.0001	<0.0001
Sodium (mg/1000 kcal)	1446.04 ± 3.12	1593.48 ± 9.92	<0.0001	<0.0001	1459.59 ± 2.37	1602.03 ± 7.85	<0.0001	<0.0001	1455.08 ± 1.94	1599.33 ± 5.28	<0.0001	<0.0001
Folate (μg/1000 kcal)	114.04 ± 0.21	109.57 ± 0.57	<0.0001	<0.0001	129.83 ± 0.18	123.49 ± 0.50	<0.0001	<0.0001	124.58 ± 0.14	118.91 ± 0.38	<0.0001	<0.0001

CHO: Carbohydrate; RE: Retinol equivalents. Values are expressed as mean ± SE; ^†^ *p* value of the unadjusted data; ^§^ *p* value of the adjusted for age, household income, education level, alcohol, smoke, exercise, BMI, and daily total energy intake. Overall data was additionally adjusted for sex using general linear regression.

**Table 4:** Prevalence and odds ratio (95% CI) of metabolic syndrome and components of rural and urban Korean adults.

	Men (*n* = 53,704)			Women (*n* = 107,622)			Overall (*n* = 161,326)		
	Urban (*n =* 46,680)	Rural (*n =* 7024)	*p-*Value	Urban (*n =* 95,457)	Rural (*n =* 12,165)	*p-*Value	Urban (*n =* 142,137)	Rural (*n =* 19,189)	*p-*Value
Metabolic syndrome									
Prevalence (*n* (%)) *	12,217 (26.17)	2684 (38.21)	<0.0001	19,803 (20.75)	4951 (40.70)	<0.0001	32,020 (22.53)	7635 (39.79)	<0.0001
Model I (OR, 95% CI)	Ref.	1.75 (1.66–1.84)	<0.0001	Ref.	2.62 (2.52–2.73)	<0.0001	Ref.	2.26 (2.19–2.33)	<0.0001
Model II (OR, 95% CI)	Ref.	1.68 (1.58–1.80)	<0.0001	Ref.	1.73 (1.65–1.81)	<0.0001	Ref.	1.65 (1.59–1.72)	<0.0001
Abdominal obesity									
Prevalence (*n* (%))	13,849 (29.7)	2353 (33.5)	<0.0001	38,904 (40.8)	7566 (62.2)	<0.0001	52,753 (37.1)	9919 (51.7)	<0.0001
Model I (OR, 95% CI)	Ref.	1.19 (1.13–1.26)	<0.0001	Ref.	2.39 (2.30–2.49)	<0.0001	Ref.	1.87 (1.82–1.93)	<0.0001
Model II (OR, 95% CI)	Ref.	1.15 (1.08–1.22)	<0.0001	Ref.	1.63 (1.56–1.70)	<0.0001	Ref.	1.36 (1.32–1.41)	<0.0001
High blood pressure									
Prevalence (*n* (%))	22,669 (48.6)	3652 (52.0)	<0.0001	32,122 (33.7)	5108 (42.0)	<0.0001	54,791 (38.6)	8760 (45.7)	<0.0001
Model I (OR, 95% CI)	Ref.	1.15 (1.09–1.21)	<0.0001	Ref.	1.43 (1.37–1.48)	<0.0001	Ref.	1.32 (1.28–1.36)	<0.0001
Model II (OR, 95% CI)	Ref.	1.00 (0.94–1.06)	0.998	Ref.	0.98 (0.94–1.03)	0.421	Ref.	0.97 (0.93–1.00)	0.044
High triglyceride									
Prevalence (*n* (%))	18,082 (38.7)	3158 (45.0)	<0.0001	18,897 (19.8)	3700 (30.4)	<0.0001	36,979 (26.0)	6858 (35.7)	<0.0001
Model I (OR, 95% CI)	Ref.	1.29 (1.23–1.36)	<0.0001	Ref.	1.77 (1.70–1.85)	<0.0001	Ref.	1.56 (1.51–1.61)	<0.0001
Model II (OR, 95% CI)	Ref.	1.25 (1.18–1.33)	<0.0001	Ref.	1.28 (1.22–1.34)	<0.0001	Ref.	1.23 (1.18–1.27)	<0.0001
High blood glucose									
Prevalence (*n* (%))	15,407 (33.0)	2701 (38.5)	<0.0001	17,892 (18.7)	2800 (23.0)	<0.0001	33,299 (23.4)	5501 (28.7)	<0.0001
Model I (OR, 95% CI)	Ref.	1.27 (1.20–1.34)	<0.0001	Ref.	1.30 (1.24–1.36)	<0.0001	Ref.	1.28 (1.24–1.33)	<0.0001
Model II (OR, 95% CI)	Ref.	1.11 (1.05–1.18)	0.000	Ref.	0.92 (0.88–0.97)	0.002	Ref.	0.96 (0.95–1.02)	0.478
Low HDL cholesterol									
Prevalence (*n* (%))	8857 (19.0)	2897 (41.2)	<0.0001	29,973 (31.4)	7934 (65.2)	<0.0001	38,830 (27.3)	10,831(56.4)	<0.0001
Model I (OR, 95% CI)	Ref.	3.00 (2.84–3.16)	<0.0001	Ref.	4.10 (3.94–4.26)	<0.0001	Ref.	3.66 (3.55–3.78)	<0.0001
Model II (OR, 95% CI)	Ref.	3.11 (2.92–3.31)	<0.0001	Ref.	3.44 (3.29–3.59)	<0.0001	Ref.	3.25 (3.14–3.37)	<0.0001

Ref.: Reference category; OR: Odds ratio; CI: Confidence intervals. Metabolic syndrome: The presence of three or more of the following components, (i) Abdominal obesity: defined by Asian-Pacific guideline, waist circumference ≥90 for men and ≥80 cm for women; (ii) High blood pressure: blood pressure ≥130/85 mmHg or medication; (iii) High triglyceride: ≥150 mg/dL; (iv) High blood glucose: fasting glucose ≥100 mg/dL or medication; (v) Low HDL cholesterol: <40 for men and <50 mg/dL for women.* *p-*Values for prevalence of MetS were calculated by χ^2^ test. Model I, unadjusted data; Model II, Model I adjusted for age, household income, education level, alcohol, smoke, exercise, BMI (not adjusted for abdominal obesity OR), and daily total energy intake. Overall data was additionally adjusted for sex using logistic regression.

**Table 5:** Adjusted odds ratio (95% CI) of the metabolic syndrome according to carbohydrate and sodium consumption.

	Quartile of Carbohydrate Consumption (g/Day)				*p*-Trend ^‡^	Quartile of Sodium Consumption (mg/Day)				*p*-Trend ^‡^
	Q1	Q2	Q3	Q4		Q1	Q2	Q3	Q4	
Men (*n* = 53,704)										
*n*	9152	13,994	14,396	16,162		11,211	12,644	13,967	15,882	
Median	226.92	285.01	329.33	412.45		1142.02	1954.54	1748.86	4061.51	
Model I	Ref.	0.98 (0.92–1.04)	1.01 (0.95–1.07)	1.07 (1.01–1.13)	0.003	Ref.	1.09 (1.03–1.15)	1.13 (1.07–1.20)	1.20 (1.14–1.27)	<0.0001
Model II	Ref.	1.01 (0.94–1.08)	1.05 (0.97–1.13)	1.06 (0.95–1.18)	0.281	Ref.	1.10 (1.03–1.17)	1.13 (1.06–1.21)	1.19 (1.11–1.28)	<0.0001
Women (*n* = 107,622)										
*n*	31,179	26,338	25,936	24,169		29,120	27,688	26,365	24,449	
Median	211.04	284.55	329.58	404.83		1113.12	1940.32	2730.04	3999.38	
Model I	Ref.	1.21 (1.17–1.26)	1.14 (1.09–1.19)	1.09 (1.05–1.14)	<0.0001	Ref.	1.02 (0.98–1.06)	1.06 (1.02–1.10)	1.14 (1.10–1.19)	<0.0001
Model II	Ref.	1.21 (1.15–1.28)	1.25 (1.17–1.33)	1.37 (1.25–1.50)	<0.0001	Ref.	1.03 (0.98–1.08)	1.05 (1.00–1.10)	1.09 (1.03–1.14)	0.0004
Overall (*n* = 161,326)										
*n*	40,331	40,332	40,332	40,331		40,331	40,332	40,332	40,331	
Median	215.03	284.72	329.48	407.65		1121.41	1944.78	2736.81	4024.33	
Model I	Ref.	1.14 (1.10–1.17)	1.10 (1.07–1.14)	1.10 (1.06–1.14)	<0.0001	Ref.	1.04 (1.01–1.08)	1.08 (1.05–1.12)	1.16 (1.12–1.20)	<0.0001
Model II	Ref.	1.14 (1.09–1.18)	1.16 (1.11–1.22)	1.23 (1.15–1.32)	<0.0001	Ref.	1.05 (1.01–1.09)	1.07 (1.03–1.11)	1.11 (1.07–1.16)	<0.0001

Ref.: Reference category; OR: Odds ratio; CI: Confidence intervals. Metabolic syndrome: The presence of three or more of the following components, (i) Abdominal obesity: defined by Asian-Pacific guideline, waist circumference ≥90 for men and ≥80 cm for women; (ii) High blood pressure: blood pressure ≥130/85 mmHg or medication; (iii) High triglyceride: ≥150 mg/dL; (iv) High blood glucose: fasting glucose ≥100 mg/dL or medication; (v) Low HDL cholesterol: <40 for men and <50 mg/dL for women. Model I, adjusted for residential area; Model II, adjusted for age, residential area, household income, education level, alcohol, smoke, exercise, BMI, and daily total energy intake. ^‡^ Linear trends across categories of carbohydrate or sodium consumption were tested using the median consumption value for each category as an ordinal variable. Overall data were additionally adjusted for sex.
